# Durable Pt-Decorated NiFe-LDH for High-Current-Density Electrocatalytic Water Splitting Under Alkaline Conditions

**DOI:** 10.3390/nano15211683

**Published:** 2025-11-06

**Authors:** Luan Liu, Hongru Liu, Baorui Jia, Xuanhui Qu, Mingli Qin

**Affiliations:** 1 Institute for Advanced Materials and Technology, University of Science and Technology Beijing, Beijing 100083, Chinaquxh@ustb.edu.cn (X.Q.); 2 Department of Materials Science and Engineering, National University of Singapore, Singapore 117575, Singapore; 3 Shunde Innovation School, University of Science and Technology Beijing, Foshan 301811, China; 4 Beijing Advanced Innovation Center for Materials Genome Engineering, University of Science and Technology Beijing, Beijing 100083, China; 5 Institute of Materials Intelligent Technology, Liaoning Academy of Materials, Shenyang 110167, China

**Keywords:** Pt clusters, NiFe-LDH, bifunctional electrocatalyst, hydrogen evolution, oxygen evolution, overall water splitting

## Abstract

The development of durable and efficient catalysts capable of driving both hydrogen and oxygen evolution reactions is essential for advancing sustainable hydrogen production through overall water electrolysis. In this study, we developed a corrosion-mediated approach, where Ni ions originate from the self-corrosion of the nickel foam (NF) substrate, to construct Pt-modified NiFe layered double hydroxide (Pt-NiFeO_x_H_y_@NiFe-LDH) under ambient conditions. The obtained catalyst exhibits a hierarchical architecture with abundant defect sites, which favor the uniform distribution of Pt clusters and optimized electronic configuration. The Pt-NiFeO_x_H_y_@NiFe-LDH catalyst, constructed through the interaction between Pt sites and defective NiFe layered double hydroxide (NiFe-LDH), demonstrates remarkable hydrogen evolution reaction (HER) activity, delivering an overpotential as low as 29 mV at a current density of 10 mA·cm^−2^ and exhibiting a small tafel slope of 34.23 mV·dec^−1^ in 1 M KOH, together with excellent oxygen evolution reaction (OER) performance, requiring only 252 mV to reach 100 mA·cm^−2^. Moreover, the catalyst demonstrates outstanding activity and durability in alkaline seawater, maintaining stable operation over long-term tests. The Pt-NiFeO_x_H_y_@NiFe-LDH electrode, when integrated into a two-electrode system, demonstrates operating voltages as low as 1.42 and 1.51 V for current densities of 10 and 100 mA·cm^−2^, respectively, and retains outstanding stability under concentrated alkaline conditions (6 M KOH, 70 °C). Overall, this work establishes a scalable and economically viable pathway toward high-efficiency bifunctional electrocatalysts and deepens the understanding of Pt-LDH interfacial synergy in promoting water-splitting catalysis.

## 1. Introduction

In the context of escalating energy demands and environmental challenges, hydrogen energy, as a representative of new energy, is of great significance to the sustainable development of society [1,2,3]. As a promising hydrogen production technology, electrocatalytic water splitting effectively overcomes the drawbacks of fossil fuel reforming that depend on finite energy sources and generate greenhouse gases [4,5,6]. However, the high energy barrier of electrocatalytic hydrogen generation enhances the cost of hydrogen energy [7,8]. Commercial Pt/C and RuO_2_ are widely acknowledged as benchmark catalysts for hydrogen and oxygen evolution (HER and OER) processes; however, their prohibitive cost continues to impede large-scale deployment [9,10,11]. Transition-metal-based compounds, including sulfides [12], carbides [13,14], phosphides [15], nitrides [16], chalcogenides [17,18], oxides [19], and hydroxides [20], have emerged as promising non-noble catalysts for the hydrogen and oxygen evolution reactions [21,22,23]. Their dual catalytic capability toward both half-reactions renders them attractive as bifunctional electrocatalysts. Designing such materials with low energy barriers and fast interfacial kinetics is of great significance for practical and large-scale hydrogen production [24,25,26,27].

However, due to the unsatisfactory HER activity of LDH materials, particularly under alkaline conditions, various modification strategies such as heteroatom incorporation, interfacial modulation [28,29], and electronic structure tuning [30] have been proposed to enhance conductivity, expose more active sites, and facilitate water dissociation kinetics, resulting in significant improvements in catalytic efficiency [31,32,33]. As a representative Pt-based catalyst, despite the nearly full atomic utilization and superior mass activity of Pt single-atom catalysts (SACs), their high-valence Pt centers tend to weaken hydrogen adsorption, thereby limiting HER efficiency [2,3]. In addition, the complexity of multistep synthesis and the demanding reaction environment significantly constrain their scalability and practical deployment [34]. Cl^−^ promotes the formation of LDHs under mild conditions with its unique corrosion performance, and the reacted material is rich in defects. Oxygen-defect-rich supports facilitate electron migration from the oxide matrix to the metal centers, tailoring the local electronic environment of catalytic sites and promoting electron accumulation beneficial for activity [35]. Moreover, metal clusters have attracted considerable attention as highly active catalysts, displaying remarkable selectivity and reactivity in diverse catalytic reactions owing to their unique structural and electronic properties [4,5]. Consequently, coupling defect-enriched LDHs with Pt clusters is predicted to facilitate highly efficient water splitting under high-current-density operation.

A practical and straightforward route was introduced to synthesize Pt clusters stabilized on defective NiFe-LDH, which involves Cl^−^-mediated corrosion during coprecipitation followed by a soaking procedure. Consequently, the cooperative optimization of the electronic structure, hierarchical quasi-aligned framework, and dense distribution of active sites jointly enhances the catalytic performance. The Pt-NiFeO_x_H_y_@NiFe-LDH catalyst demonstrates outstanding hydrogen evolution activity, delivering 10 mA·cm^−2^ at an overpotential of only 29 mV and exhibiting excellent OER performance with 252 mV required to reach 100 mA·cm^−2^. Owing to its superior bifunctional activity, the Pt-NiFeO_x_H_y_@NiFe-LDH electrolyzer sustains current outputs of 10 and 100 mA·cm^−2^ at remarkably low operational voltages of 1.42 and 1.51 V in alkaline environments, demonstrating its potential for practical applications. This research further establishes a versatile platform to clarify the function of Pt clusters embedded in defective NiFe-LDH nanosheets toward boosting electrocatalytic efficiency.

## 2. Results and Discussion

### 2.1. Synthesis and Characterization

**Figure 1:**
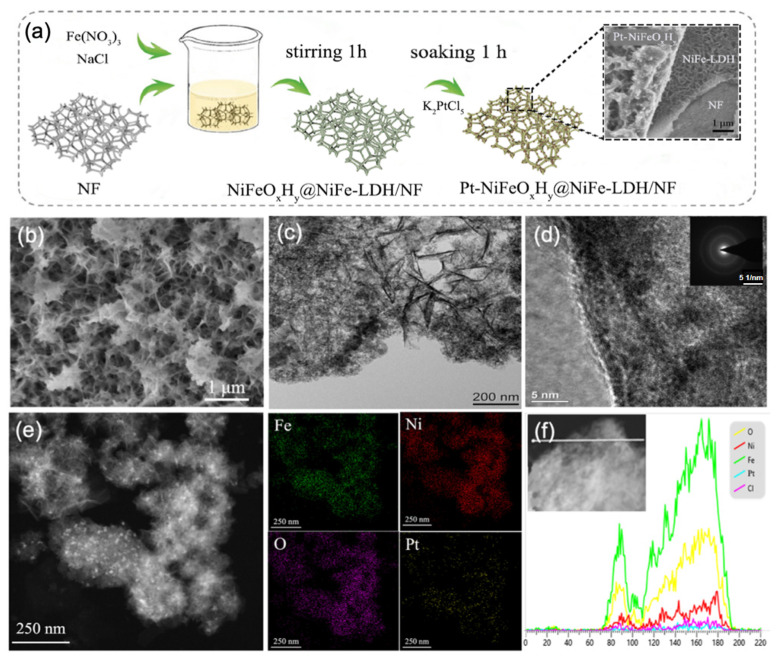
Structural and morphological analysis of Pt-NiFeO_x_H_y_@NiFe-LDH: (**a**) schematic diagram of the synthesis procedure (the Ni source comes from Ni Foam), (**b**) SEM micrographs, (**c**) dark-field TEM image, (**d**) high-resolution TEM image, (**e**) elemental mapping and (**f**) cross-sectional EDS line-scan profiles.

The process of growing composite Pt-NiFeO_x_H_y_@NiFe-LDH on the nickel foam (NF) substrate by corrosion strategy is shown in Figure 1a. NiFe-LDH can be rapidly formed within 10 min, and NiFeO_x_H_y_ continues to grow on the structure subsequently. Notably, the synthesis was accomplished under ambient conditions without harsh treatments. XRD analysis of Pt-NiFeO_x_H_y_@NiFe-LDH/NF (Figure S1) revealed only the diffraction peaks of Ni foam, suggesting that the as-prepared materials are amorphous.

SEM analysis of the Pt-NiFeO_x_H_y_@NiFe-LDH (Figure 1b) illustrates that the NF substrate is homogeneously coated with a NiFe-LDH layer, while amorphous filament-like structures coalesce into uniform spherical aggregates. In contrast to the initially smooth NF surface, the corroded samples exhibit a distinctly altered morphology, confirming the formation of new phases (Figure S2). The enlarged-TEM images (Figure 1c) show that the thickness of these NiFe-LDH nanosheets is 5–20 nm, and their lateral size is over 200 nm. The heterogeneous interface of NiFeO_x_H_y_ and NiFe-LDH can be clearly seen in the high-resolution TEM image in Figure 1d.

The SAED pattern of Pt-NiFeO_x_H_y_@NiFe-LDH (inset in Figure 1d) further confirms its amorphous nature, as the presence of a diffuse halo ring is consistent with the XRD results (Figure S1), and minor features are attributable to the Ni foam and background. HAADF-STEM image (Figure 1e) reveals that Pt nanoparticles are homogeneously dispersed as bright dots over the NiFeO_x_H_y_ surface. This is attributed to the fact that the abundant defect sites possessed by the amorphous filamentary structures of NiFeO_x_H_y_@NiFe-LDH are favorable for the loading of Pt. The amorphous filamentary structure of NiFeO_x_H_y_@NiFe-LDH (Figure S3) is favorable for the loading of Pt. The elemental mapping images of Pt-NiFeO_x_H_y_@NiFe-LDH (Figure 1e) show that Ni, Fe, O, and Pt elements are non-uniformly distributed, further confirming the successful incorporation of Pt species. And the dense distribution of Ni and Cl on NiFe-LDH can be clearly seen, which is consistent with the inference that Ni concentration affects crystal formation and Cl acts as an anion intercalation to maintain the LDH phase. As depicted in Figure 1f, the cross-sectional EDS line profiles indicate that Fe exhibits a stronger signal than Ni, especially near the amorphous edge region.

### 2.2. Electrocatalyst Characterizations

#### 2.2.1. Hydrogen Evolution Reaction Performance

**Figure 2:**
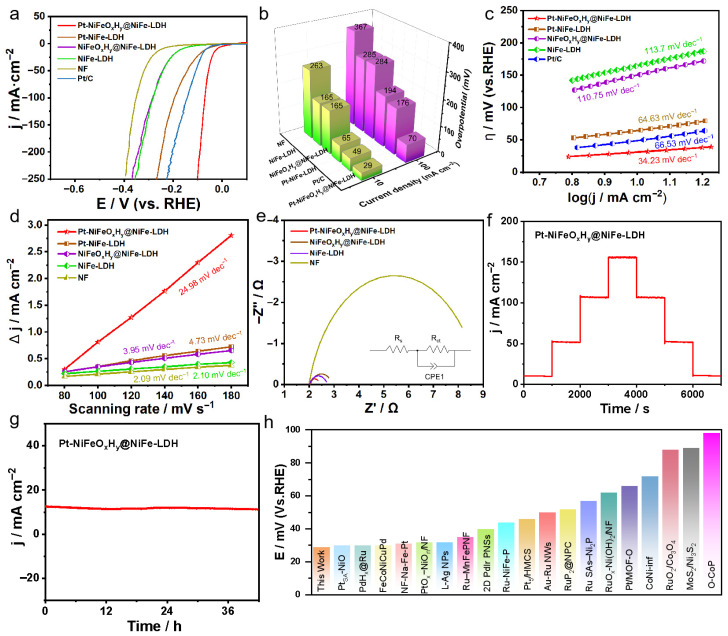
Electrochemical evaluation of HER performance: (**a**) LSV profiles (with 95% iR-corrected), (**b**) comparison of overpotentials for the synthesized catalysts at 10 and 100 mA·cm^−2^, (**c**) Tafel slop, (**d**) electrochemical surface area, (**e**) EIS spectra, (**f**) multi-step chronopotentiometric measurements, (**g**) long-term stability evaluation of Pt-NiFeO_x_H_y_@NiFe-LDH via chronopotentiometry, (**h**) comparison of the overpotentials measured at 10 mA·cm^−2^ with benchmark catalysts reported in 1 M KOH clearly indicates the superior HER performance of Pt-NiFeO_x_H_y_@NiFe-LDH.

Given the intrinsically sluggish hydrogen-evolution kinetics of NiFe-LDH, improving its catalytic performance remains a critical task. The as-prepared Pt-NiFeO_x_H_y_@NiFe-LDH electrode, operated as an integrated catalyst, was examined for its hydrogen evolution performance under room-temperature conditions within a standard three-electrode configuration. Figure 2a shows the LSV curves obtained in 1 M KOH after 95% iR correction. The optimized Pt-NiFeO_x_H_y_@NiFe-LDH exhibits remarkable HER performance—reaching 10 mA·cm^−2^ at an overpotential as low as 29 mV—compared to the other control samples of Pt/C (49 mV@10 mA·cm^−2^), Pt-NiFe-LDH (65 mV@10 mA·cm^−2^), NiFeO_x_H_y_@NiFe-LDH (165 mV@10 mA·cm^−2^), NiFe-LDH (165 mV@10 mA·cm^−2^), and NF (263 mV@10 mA·cm^−2^), highlighting the essential role of Pt and the synergy effect in enhancing HER efficiency (Figure 2b).

To further probe the catalytic kinetics, the Tafel slopes were analyzed. Figure 2c demonstrates that Pt-NiFeO_x_H_y_@NiFe-LDH delivers a smaller slope of 34.23 mV·dec^−1^ compared with the references, indicative of accelerated reaction kinetics beneficial for HER. The superior intrinsic HER performance of Pt-NiFeO_x_H_y_@NiFe-LDH is corroborated by its higher double-layer capacitance (C_dl_), as determined from CV tests (Figure S4). As presented in Figure 2d, Pt-NiFeO_x_H_y_@NiFe-LDH exhibits outstanding C_dl_ value of 24.98 mF·cm^−2^ compared with Pt-NiFe-LDH (4.73 mF·cm^−2^), NiFeO_x_H_y_@NiFe-LDH (3.95 mF·cm^−2^), NiFe-LDH (2.10 mF·cm^−2^) and NF (2.09 mF·cm^−2^), confirming that Pt-NiFeO_x_H_y_@NiFe-LDH exhibits an increased density of catalytically active sites. In addition to catalytic activity, durability is a crucial factor for an efficient HER catalyst.

As illustrated in Figure 2e, the impedance characteristics recorded for various catalysts in 0.5 M electrolyte reveal clear distinctions. The Pt-NiFeO_x_H_y_@NiFe-LDH sample exhibits a notably reduced semicircle diameter, suggesting a lower charge-transfer resistance (R_ct_), which promotes faster interfacial electron migration and enhances overall reaction kinetics. In contrast, NiFeO_x_H_y_@NiFe-LDH and NiFe-LDH show relatively larger semicircles, while the bare NF electrode exhibits the largest impedance, suggesting the slowest charge-transfer process. The equivalent circuit fitting further confirms the superior conductivity and charge-transfer capability of Pt-NiFeO_x_H_y_@NiFe-LDH at the electrochemical interface. For practical use, catalyst stability was evaluated by multi-step chronopotentiometry.

As illustrated in Figure 2f, Pt-NiFeO_x_H_y_@NiFe-LDH delivers stable potential responses across different current densities, maintaining reversibility throughout the process. Notably, Pt-NiFeO_x_H_y_@NiFe-LDH shows negligible performance loss during 42 h of continuous operation at a fixed potential in 1 M KOH (Figure 2g), confirming its outstanding durability. These findings further demonstrate the material’s structural robustness and long-term stability. The electrochemical performance of Pt-NiFeO_x_H_y_@NiFe-LDH surpasses that of the majority of previously reported metal-based electrocatalysts (Figure 2h and Table S1). The Pt-NiFeO_x_H_y_@NiFe-LDH catalyst exhibits superior electrochemical behavior compared with most of the metal-based electrocatalysts reported to date (Figure 2h and Table S1).

About 97% of the Earth’s water resources are seawater, leaving only ~3% as freshwater. Given its abundance, seawater electrolysis is considered a promising route to mitigate the energy crisis. Accordingly, the HER activities of Pt-NiFeO_x_H_y_@NiFe-LDH, together with NF, NiFeO_x_H_y_, NiFe-LDH, NiFeO_x_H_y_@NiFe-LDH, and Pt-NiFe-LDH, were evaluated in alkaline seawater sampled from the coastal region of the Yellow Sea in China under identical conditions.

**Figure 3:**
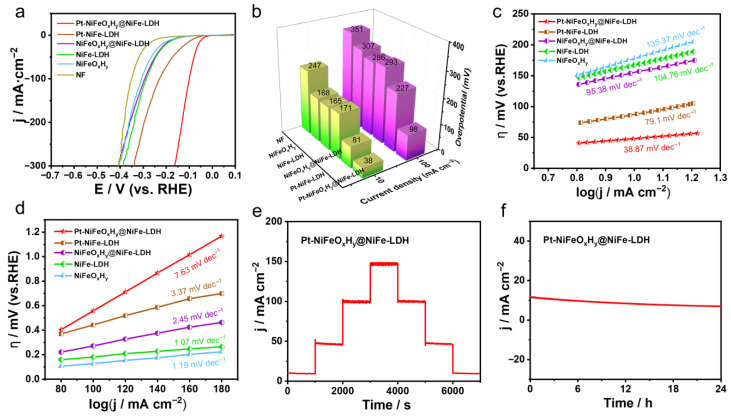
Comprehensive characterization of the HER performance of Pt-NiFeO_x_H_y_@NiFe-LDH in 1 M KOH seawater, including (**a**) polarization curves (with 95% iR-corrected), (**b**) comparison of overpotentials at 10 and 100 mA·cm^−2^, (**c**) Tafel plots, (**d**) determination of the electrochemically active surface area, (**e**) stepwise chronopotentiometric profiles, and (**f**) long-term durability evaluation.

Figure 3a displays the LSV curves, where the Pt-NiFeO_x_H_y_@NiFe-LDH catalyst demonstrates outstanding hydrogen evolution performance in alkaline seawater, showing overpotentials as low as 38 and 98 mV at current densities of 10 and 100 mA·cm^−2^, respectively, underscoring its remarkable catalytic efficiency and strong potential for practical seawater electrolysis. This result confirms its excellent catalytic behavior after corrosion. A similar trend is further illustrated by the overpotential comparison at 10 and 100 mA·cm^−2^ in Figure 3b.

The Pt-NiFeO_x_H_y_@NiFe-LDH catalyst exhibits the smallest Tafel slope of 38.87 mV·dec^−1^ among all reference samples, indicating fast reaction kinetics toward HER in 1 M KOH seawater (Figure 3c). Moreover, the excellent HER activity of Pt-NiFeO_x_H_y_@NiFe-LDH is further confirmed by its larger C_dl_ calculated from CV measurements (Figure S5).

As presented in Figure 3d, Pt-NiFeO_x_H_y_@NiFe-LDH possesses a larger C_dl_ value of 7.63 mF·cm^−2^. This implies that Pt-NiFeO_x_H_y_@NiFe-LDH exposes more active sites than the other samples. Furthermore, its stability in alkaline seawater was examined using the same procedure as in 1 M KOH. As shown in Figure 3e, multi-step chronopotentiometry reveals stable potential responses at different current densities, with consistent reversibility throughout the process. Especially, Pt-NiFeO_x_H_y_@NiFe-LDH catalyst maintains a stable potential with negligible decay over 24 h of continuous operation at a fixed bias, confirming its outstanding durability in alkaline seawater (Figure 3f).

#### 2.2.2. Oxygen Evolution Reaction Performance

Since the oxygen evolution reaction suffers from slow kinetics, designing efficient OER catalysts is vital for hydrogen production. To this end, Pt-NiFeO_x_H_y_@NiFe-LDH, together with control samples, were examined in 1 M KOH employing the same three-electrode system. According to the polarization plots, Pt-NiFeO_x_H_y_@NiFe-LDH delivers the highest oxygen evolution activity compared with all reference samples.

**Figure 4:**
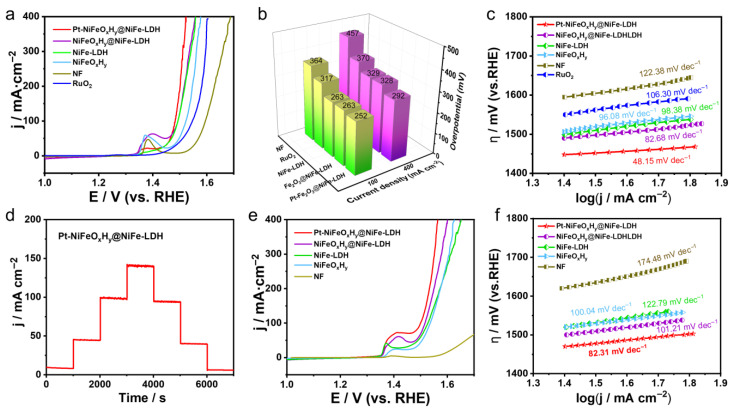
Electrochemical OER tests: (**a**) LSV curves (with 95% iR-corrected), (**b**) Comparison of overpotentials at 100 and 400 mA·cm^−2^ for different samples, (**c**) Tafel slope analysis, (**d**) Stepwise chronopotentiometry measurement, (**e**,**f**) LSV results (with 95% iR-corrected) and Tafel plots assessment in 1 M KOH seawater.

Figure 4a,b illustrate that Pt-NiFeO_x_H_y_@NiFe-LDH requires only 252 mV and 292 mV to achieve current densities of 100 and 400 mA·cm^−2^, respectively, which exceeds the performance of RuO_2_ and all other tested samples. It is demonstrated that the NiFeO_x_H_y_@LDH structure retains the excellent electrocatalytic features of LDH, while the enhanced activity mainly originates from the strong interfacial charge coupling between Pt oxides and the underlying metal support, which facilitates charge transfer and thereby boosts the overall catalytic efficiency. Moreover, PtO_x_ catalysts effectively lower the reaction barrier and provide plentiful surface sites that promote the oxygen evolution process. In agreement with previous studies [36], interfacial oxygen atoms bridge the PtO_x_ phase and neighboring transition-metal sites, leading to electronic redistribution and the formation of a structurally stable Pt-O-Ni linkage, serving to immobilize nanoparticles and reinforce the cooperative behavior between catalyst and support.

Such interfacial stabilization enhances the synergistic contribution of Pt oxides with the host material, thereby delivering OER activity surpassing that of NiFeO_x_H_y_@LDH. Figure 4c reveals that Pt-NiFeO_x_H_y_@NiFe-LDH possesses the smallest Tafel slope (48.15 mV·dec^−1^), reflecting accelerated reaction kinetics for oxygen evolution. 

Such high activity arises from the cooperative effect of multiple constituents together with plentiful surface-active sites. The durability tests of Pt-NiFeO_x_H_y_@NiFe-LDH are performed. Figure 4d presents the multi-step chronoamperometric profile, where the current density increases with potential and remains stable during both forward and reverse sweeps, suggesting strong stability. In Figure S6, Pt-NiFeO_x_H_y_@NiFe-LDH demonstrates excellent durability throughout 42 h of OER operation.

Moreover, its robust structure enables efficient oxygen evolution even in alkaline seawater electrolyte under identical testing conditions. As depicted in Figure 4e, Pt-NiFeO_x_H_y_@NiFe-LDH demonstrates high oxygen evolution activity in alkaline seawater, achieving 10 and 100 mA·cm^−2^ at overpotentials of only 290 and 460 mV. Furthermore, its small Tafel slope of 82.31 mV·dec^−1^ (Figure 4f) confirms favorable kinetics toward OER in 1 M KOH seawater. In alkaline seawater, Cl^−^ corrosion and competing chlorine evolution reaction can deteriorate OER on bare NF, leading to higher overpotentials; however, the Pt-NiFeO_x_H_y_@NiFe-LDH interface mitigates these effects.

As shown in Figure S7, the catalyst sustained constant current output for 42 h under extended durability evaluation in alkaline seawater. Collectively, these electrochemical findings demonstrate the superior HER activity of PtO_x_ and also show that the NiFe-LDH component predominantly facilitates OER kinetics, whereas the introduction of Pt species imparts bifunctional catalytic behavior to the Pt-NiFeO_x_H_y_@NiFe-LDH, enabling efficient overall water splitting with active centers that participate in both HER and OER processes.

#### 2.2.3. Overall Water-Splitting Performance

**Figure 5:**
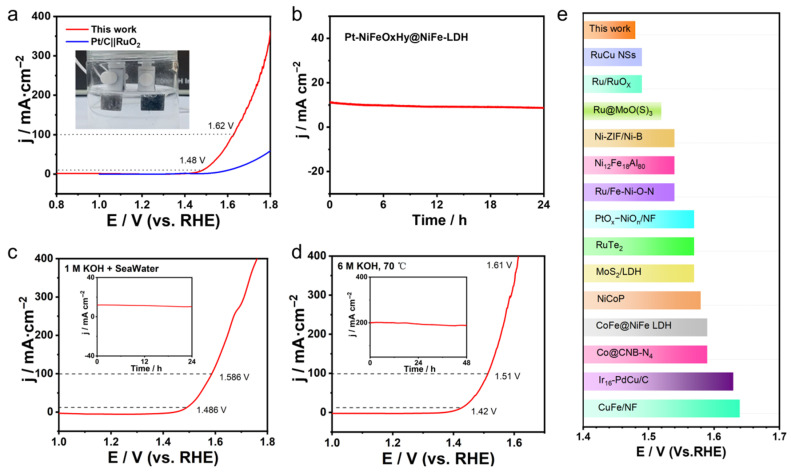
(**a**) LSV curves of Pt-NiFeO_x_H_y_@NiFe-LDH||Pt-NiFeO_x_H_y_@NiFe-LDH for overall water splitting (with 95% iR-corrected) (inset: photograph of the two-electrode configuration), (**b**) Chronoamperometry evaluation conducted in 1 M KOH electrolyte, (**c**) LSV profiles recorded for overall water splitting in a 1 M KOH-seawater electrolyte (with 95% iR-corrected), (**d**) LSV profiles measured in 6 M KOH at 70 °C (with 95% iR-corrected), the inset in (**c**,**d**) shows the stability assessment of overall water-splitting performance, (**e**) Comparison of overpotentials with data from recent literature.

Inspired by the outstanding bifunctional electrocatalytic properties of Pt-NiFeO_x_H_y_@NiFe-LDH, an integrated two-electrode electrolyzer was constructed for overall water splitting, employing Pt-NiFeO_x_H_y_@NiFe-LDH as both electrodes in an alkaline (1 M KOH) environment. As depicted in Figure 5a, the electrolyzer attains current densities of 10 and 100 mA·cm^−2^ at applied cell voltages of 1.48 and 1.58 V, respectively, demonstrating excellent overall water-splitting efficiency. The inset of Figure 5a displays a photograph of the operating cell, where vigorous bubble generation can be clearly observed on both electrode surfaces.

It is noteworthy that the Pt-NiFeO_x_H_y_@NiFe-LDH exhibits better overall water-splitting efficiency than the benchmark Pt/C‖RuO_2_ and other recently reported catalysts under alkaline conditions (Figure 5c and Table S2). Furthermore, chronoamperometry indicates that the electrodes can continuously sustain constant current output for 24 h, highlighting their durability (Figure 5b). Notably, the electrolyzer achieves outstanding water-splitting performance when operated in alkaline seawater. As presented in Figure 5d,e, current densities of 10 and 100 mA·cm^−2^ are obtained at low voltages of 1.48 and 1.62 V, respectively, with excellent stability.

In addition, the Pt-NiFeO_x_H_y_@NiFe-LDH device has been further examined under harsh operating environments. In 6 M KOH at 70 °C, Pt-NiFeO_x_H_y_@NiFe-LDH demonstrates superior catalytic behavior, suggesting potential for real-world operation. More importantly, the electrolyzer maintains 200 mA·cm^−2^ for at least 48 h under constant potential (the inset of Figure 5d), highlighting its robust stability. The remarkable activity and durability can be attributed to the synergistic composition of the catalyst. First, high electrical conductivity and effective mass diffusion of Pt-NiFeO_x_H_y_@NiFe-LDH are attributed to the conductive NF support and the robust interfacial coupling between the catalyst and the substrate.

Secondly, the large electrochemical surface area reflected by the high C_dl_ value indicates numerous accessible active sites, giving Pt-NiFeO_x_H_y_@NiFe-LDH superior intrinsic HER/OER activity over the reference samples. More importantly, the enhanced catalytic behavior stems from the synergy between PtO_x_ and NiFeO_x_H_y_@LDH, which facilitates the dissociation of OH^−^ and H_2_O.

## 3. Conclusions

In summary, a corrosion-assisted coprecipitation strategy was developed to construct Pt cluster-decorated NiFeO_x_H_y_@LDH nanosheets on nickel foam. The unique architecture, featuring abundant defect sites and uniform Pt dispersion, endows the catalyst with outstanding bifunctional activity. In alkaline electrolytes, Pt-NiFeO_x_H_y_@NiFe-LDH achieves current densities of 10 and 100 mA·cm^−2^ at low overpotentials of 29 and 252 mV for the HER and OER, respectively, outperforming most state-of-the-art non-noble-metal catalysts. In addition, the catalyst demonstrates outstanding long-term stability and persistent activity under alkaline seawater conditions, while also sustaining industrial-achieving high current densities at relatively low operating voltages. Overall, this study establishes Pt-NiFeO_x_H_y_@NiFe-LDH as a robust and intrinsically active bifunctional electrocatalyst capable of sustaining overall water splitting under industrially relevant conditions and provides fundamental insights into the cooperative electronic coupling between Pt clusters and defect-engineered LDH layers that governs its superior catalytic performance.

## Data Availability

The data presented in this study are available in the article and Supplementary Materials.

## Supplementary Materials

The following supporting information can be downloaded at: https://www.mdpi.com/article/10.3390/nano15211683/s1, Figure S1: XRD patterns of Pt-NiFeO_x_H_y_@NiFe-LDH/NF before and after HER test. Figure S2: SEM pictures of NiFeO_x_H_y_@NiFe-LDH at different stirring times. Figure S3: SEM image and corresponding EDS elemental mapping images of NiFeO_x_H_y_@NiFe-LDH. Figure S4: Current density of cyclic voltammograms against scan rates of different catalytic electrodes within a potential range where no Faradaic process is observed in 1M KOH. Figure S5: Current density of cyclic voltammograms against scan rates of different catalytic electrodes within a potential range where no Faradaic process is observed in 1M KOH with seawater. Figure S6: Results of the stability test for Pt-NiFeOxHy@NiFe-LDH in 1 M KOH. Figure S7: Results of the stability test for Pt-NiFeOxHy@NiFe-LDH in 1 M KOH seawater. Table S1: Comparison of the electrocatalytic activity of Pt-NiFeOxHy@NiFe-LDH in 1M KOH electrolyte with some HER cata-lysts. Table S2: Comparison of the electrocatalytic activity of Pt-NiFeO_x_H_y_@NiFe-LDH in 1M KOH electrolyte with some OER catalysts.
