# Supplementary material for: Durable Pt-Decorated NiFe-LDH for High-Current-Density Electrocatalytic Water Splitting Under Alkaline Conditions
Source: Nanomaterials (Basel). 2025 Nov 6;15(21):1683. doi: 10.3390/nano15211683 (PMC12609545; doi:10.3390/nano15211683)
Supplement: Supplementary file 1 [file nanomaterials-15-01683-s001.zip › nanomaterials-3918406-supplementary .pdf]

Supporting Information

Durable Pt-Decorated NiFe-LDH for High-Current-Density Electrocatalytic Water Splitting Under Alkaline Conditions

Luan Liu [1,*,†], Hongru Liu [1,†], Baorui Jia [1,2,3,*], Xuanhui Qu [1,4] and Mingli Qin [1,4,5,*]

[1] Institute for Advanced Materials and Technology, University of Science and Technology Beijing, Beijing 100083,China; quxh@ustb.edu.cn (X.Q.)
[2] Department of Materials Science and Engineering, National University of Singapore, Singapore 117575, Singapore
[3] Shunde Innovation School, University of Science and Technology Beijing, Foshan 301811, China
[4] Beijing Advanced Innovation Center for Materials Genome Engineering, University of Science and Technology Beijing, Beijing 100083, China
[5] Institute of Materials Intelligent Technology, Liaoning Academy of Materials, Shenyang 110167, China
* Correspondence: 527708682@qq.com (L.L.); jiabaorui@ustb.edu.cn (B.J.); qinml@mater.ustb.edu.cn (M.Q.)
† These authors contributed equally to this work.

1 Experimental section

1.1 Materials

Ferric nitrate nonahydrate ($Fe(NO_3)_3·9H_2O$, ≥98%) and sodium chloride (NaCl, ≥99%) were purchased from Shanghai Aladdin Biochemical Technology Co., Ltd. (China). Potassium tetrachloroplatinate(II) (K_2PtCl_4, ≥99%) was purchased from Macklin Biochemical Co., Ltd. (Shanghai, China). Ethanol (≥99.7%) and deionized (DI) water were purchased from Sinopharm Chemical Reagent Co., Ltd. (Shanghai, China). All reagents were of analytical grade and used without further purification. Deionized (DI) water was used throughout the synthesis and washing processes, and ethanol (≥99.7%) was employed for cleaning and rinsing the samples. Nickel foam (NF, 1.0 mm thickness, porosity 95%, 99.9% purity) was purchased from Suzhou Keshenghe Metal Materials Co., Ltd.

(China). The seawater used was collected from the Yellow Sea of China and prepared as a 1 M KOH solution without removing halides in order to maintain the authenticity of the chloride content. All seawater tests were carried out using the same electrolyzer configuration.

1.2 Catalyst synthesis

1.2.1 Synthesis of $NiFeO_xH_y$@NiFe-LDH

Nickel foam (NF) was first treated with ultrasonic technology for 15 minutes in diluent hydrochloric acid, ethanol, and deionized water in order to remove the oxides. The $NiFeO_xH_y$@NiFe-LDH catalyst was prepared via a simple one-step method under ambient conditions. One piece of Ni foam (1 × 1.5 cm^2) was immersed in 1 mmol NaCl and 4 mmol $Fe(NO_3)_3·9H_2O$ solution and stirred at room temperature for 1 h. After the reaction, the product was dried at 60 °C for 3 h to form $NiFeO_xH_y$@NiFe-LDH. The NiFe-LDH was fabricated similarly to $NiFeO_xH_y$@NiFe-LDH, only being stirred for 10 min. In addition, contrast samples with different reactant concentrations and reaction times were also synthesized.

1.2.2 Synthesis of Pt-$NiFeO_xH_y$@NiFe-LDH

Pt modification was carried out by immersing the $NiFeO_xH_y$@NiFe-LDH sample in ethanol solution containing 5 $mg·mL^{-1}$ potassium chloroplatinite for

1 hour, followed by washing and drying. The final Pt loading, determined by ICP analysis, shows a Pt mass ratio of 0.62%.

1.2.3 Synthesis of $NiFeO_xH_y$@NiFe-LDH

The $NiFeO_xH_y$@NiFe-LDH catalyst was prepared in the presence of 1mmol NaCl and 4 mmol $Fe(NO_3)_3{\cdot}9H_2O$ on Ni Foam (1 × 1.5 cm^2) and stirred for 1 h at room temperature. After the reaction, the product was dried at 60 °C for 3 h to form $NiFeO_xH_y$@NiFe-LDH.

2.1 Characterizations

The phases were characterized using XRD on a Rigaku D/max-RB12 diffractometer, with Cu Kα radiation in the 2-θ range of 10-90°. SEM was conducted using an SU8100 electron microscope. TEM, HRTEM, and EDS mapping experiments were performed on a microscope (FEI Tecnai G2F30), and the associated cross-sectional EDS compositional line profiles were obtained using the same instrument. Inductively coupled plasma mass spectrometry (ICP-MS) was performed on an Agilent cOES730 using argon as the carrier gas.

2.2 Electrochemical measurement

All the electrochemical tests were performed via an electrochemical workstation (CHI 760D) at room temperature in a typical three-electrode setup, with 1 M KOH; a graphite rod and mercury–mercury oxide electrode were used as the counter and reference electrodes, respectively. The samples prepared on Ni foam (1 cm^2) served as the working electrode. Linear sweeping voltammograms (LSVs) were studied at a scan rate of 5 mV s^{-1} with 95 % iR correction. Electrochemical impedance spectroscopy (EIS) measurements were carried out at frequency ranges from 100 kHz to 0.01 Hz with an amplitude of 5 mV. Long-term stability measurement was collected at a specific potential. In the measurement of ESCA, the CV potential window ranged from 0.85 to 0.95 vs. RHE. The scan rates were 80, 100, 120, 140, 160, and 180mV s^{-1}. C_{dl} was calculated using the following equation: j_c*A= vC_{dl}, where v is the scan rate, j_c is the current at 0.9 V vs. RHE, and A is the surface area of the NF electrode (1 cm^2). The slope of the plot of j_c*A as a function of v is equal to C_{dl}.

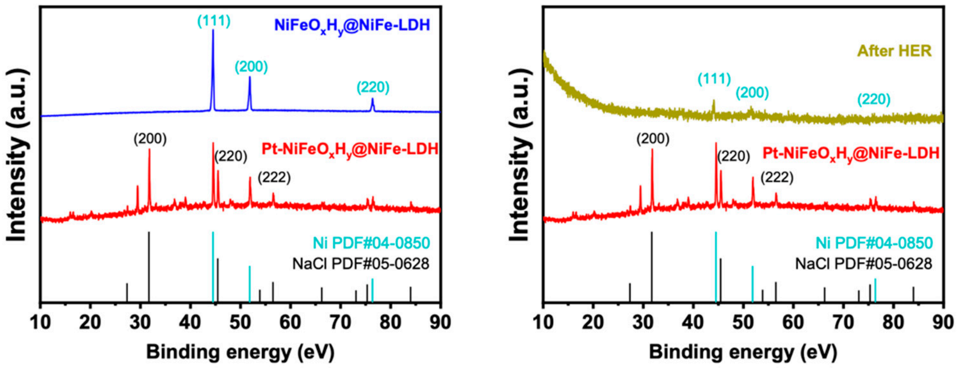

Figure S1. XRD patterns of Pt-NiFeO$_x$H$_y$@NiFe-LDH/NF before and after HER test.

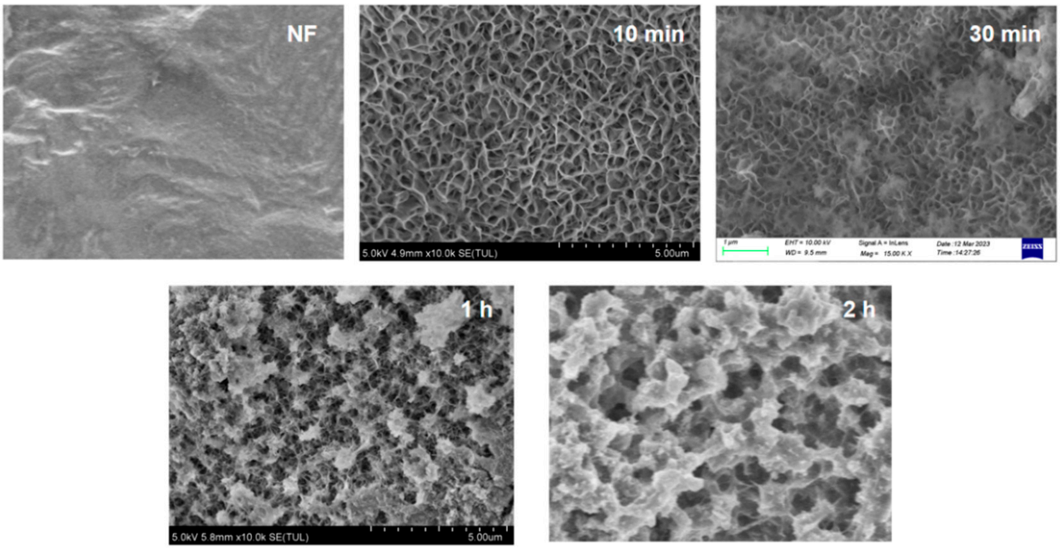

Figure S2. SEM pictures of NiFeO$_x$H$_y$@NiFe-LDH at different stirring times.

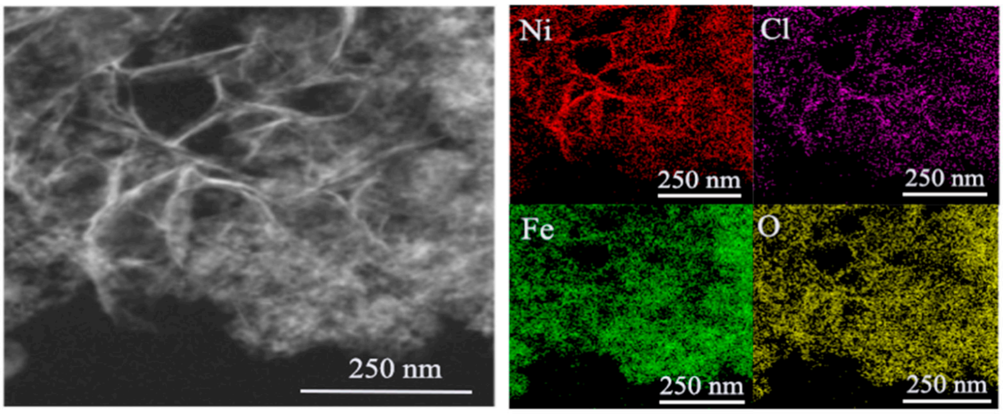

Figure S3. SEM image and corresponding EDS elemental mapping images of $NiFeO_xH_y$@NiFe-LDH.

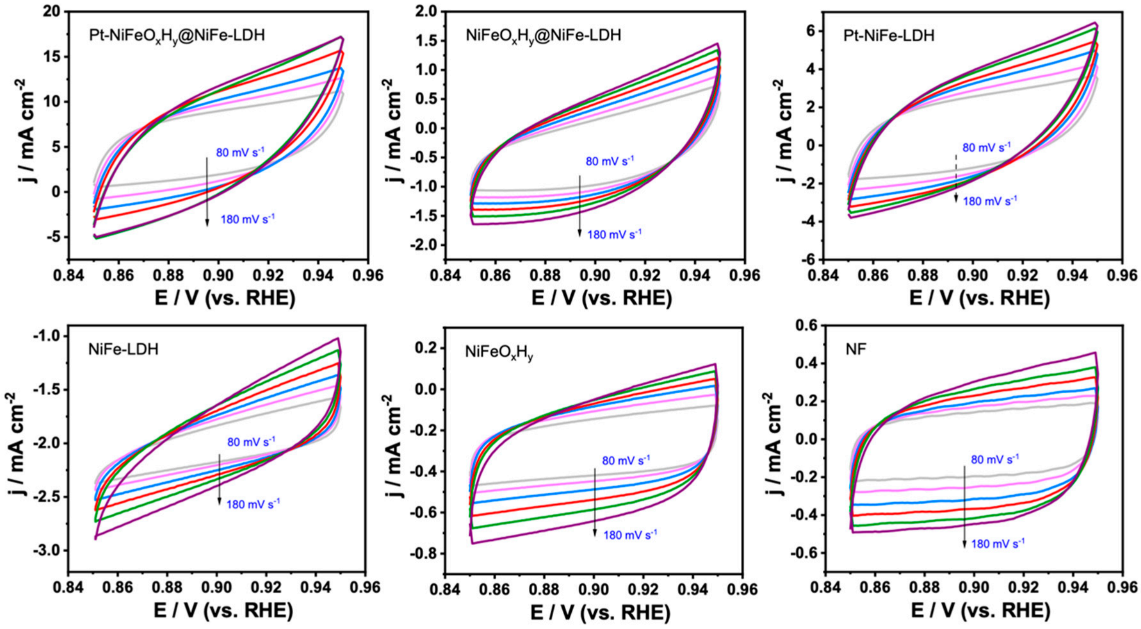

Figure S4. Current density of cyclic voltammograms against scan rates of different catalytic electrodes within a potential range where no Faradaic process is observed in 1M KOH.

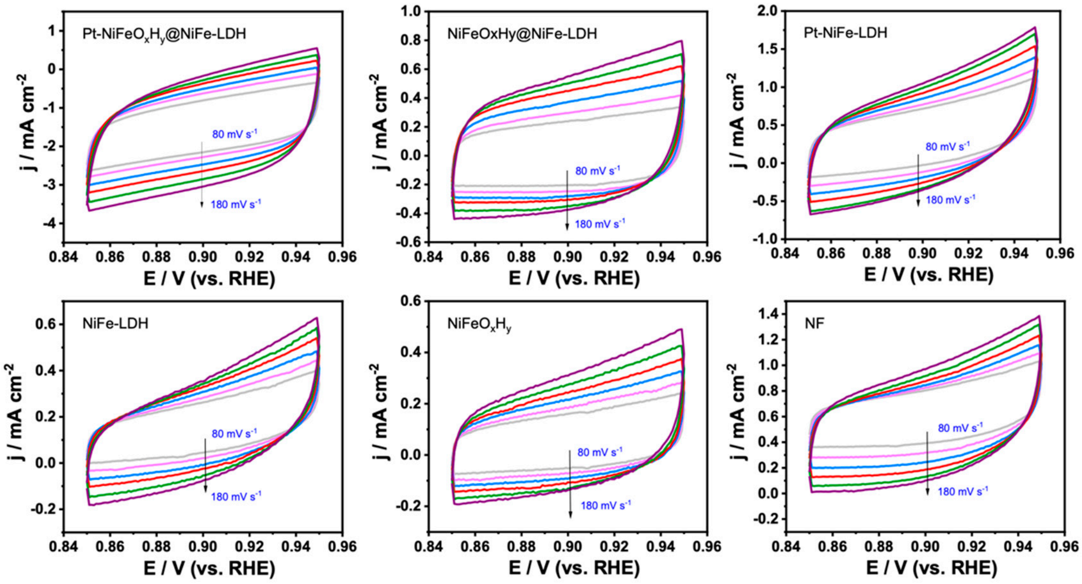

Figure S5. Current density of cyclic voltammograms against scan rates of different catalytic electrodes within a potential range where no Faradaic process is observed in 1M KOH with seawater.

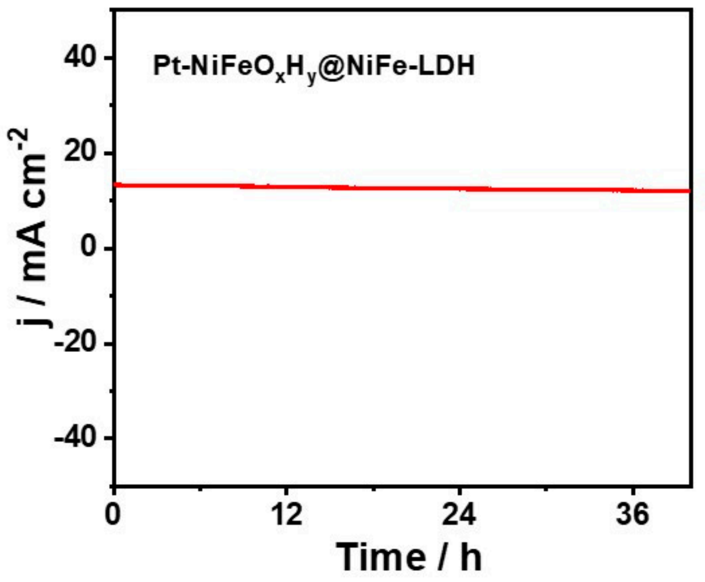

Figure S6. Results of the stability test for Pt-$NiFeO_xH_y$@NiFe-LDH in 1 M KOH.

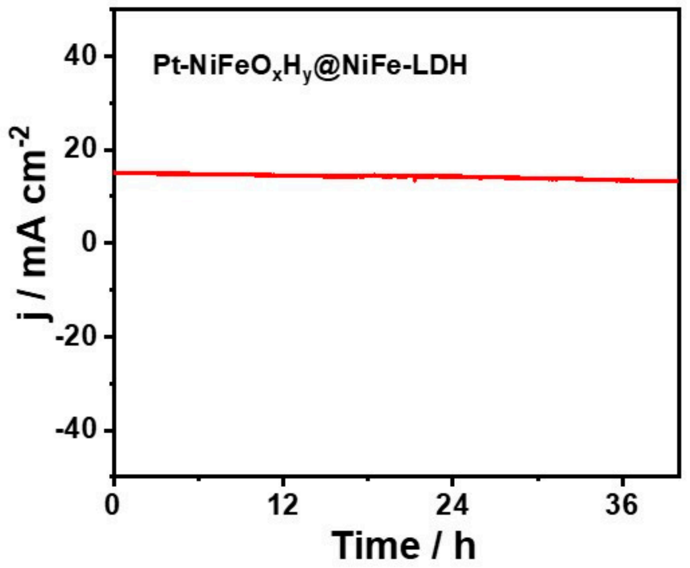

Figure S7. Results of the stability test for Pt-$NiFeO_xH_y$@NiFe-LDH in 1 M KOH seawater.

Table S1. Comparison of the electrocatalytic activity of Pt-$NiFeO_xH_y$@NiFe-LDH in 1M KOH electrolyte with some HER catalysts.

Sample	η_{10} (mV)	DOI:
This work	29	
PtSA-NiO	35	10.1038/s41467-021-24079-8
PdH_x@Ru	30	10.1021/jacs.2c11692
FeCoNiCuPd	30	10.1016/j.apcatb.2022.121472
NF-Na-Fe-Pt	31	10.1016/j.apcatb.2021.120395
PtO_x–NiOn/NF	32	10.1007/s12274-022-5369-0
L-Ag NPs	32	10.1038/s41929-019-0365-9
Ru–MnFePNF	35	10.1002/aenm.202000814
2D PdIr PNSs	40	10.1016/j.apsusc.2020.145408
Ru-NiFe-P	44	10.1016/j.apcatb.2019.118324
Pt_5/HMCS	46.2	10.1002/adma.201901349
Au-Ru NWs	50	10.1038/s41557-018-0012-0
RuP2@NPC	52	10.1002/anie.201704911
Ru SAs–Ni_2P	57	10.1016/j.nanoen.2020.105467
RuO_x-$Ni(OH)_2$/NF	62	10.1039/D4CY01074D
Pt/MOF-O	66	10.1021/jacs.1c06006
CoNi-inf	72	10.1002/adma.202002857
RuO_2/Co_3O_4	88	10.1039/C6RA25810G
MoS_2/Ni_3S_2	89	10.1002/smll.202006730
O-CoP	98	10.1002/adfm.201905252

Table S2. Comparison of the electrocatalytic activity of Pt-$NiFeO_xH_y$@NiFe-LDH in 1M KOH electrolyte with some OER catalysts.

Sample	η_{10} (mV)	DOI:
This work	1.48	
RuCu NSs	1.49	10.1002/ange.201908092
Ru/RuO_X	1.49	10.1007/s12274-021-3590-x
Ru@$MoO(S)_3$	1.52	10.1016/j.nanoen.2022.107445
Ni-ZIF/Ni-B	1.54	10.1002/aenm.201902714
$Ni_{12}Fe_{18}Al_{80}$	1.54	10.1002/anie.202300800
Ru/Fe-Ni-O-N	1.54	10.1039/D2TA07586E
PtO_x-NiO_n/NF	1.57	10.1007/s12274-022-5369-0
MoS_2/LDH	1.57	10.1021/acs.nanolett.9b01329
$RuTe_2$	1.57	10.1016/j.apcatb.2020.119281
NiCoP	1.58	10.1021/acs.nanolett.6b03803
CoFe@NiFe LDH	1.59	10.1016/j.apcatb.2019.04.054
Co@CNB-N_4	1.59	10.1016/j.cej.2023.141435
Ir16-PdCu/C	1.63	10.1021/acs.nanolett.1c01581
